# Targeting mitochondria to extend physically active lifespan

**DOI:** 10.18632/aging.204494

**Published:** 2023-01-16

**Authors:** Bumsoo Ahn

**Affiliations:** 1 Gerontology and Geriatrics section, Department of Internal Medicine, Wake Forest School of Medicine, Winston-Salem, NC 27101, USA

**Keywords:** peroxiredoxin, mitochondria, sarcopenia, neurogenic atrophy, retrograde effect

The proportion of the world’s population over 60 years of age is projected to nearly double in the next two decades, but the advances in the life span bring us new challenges, the functional deficit and impaired life quality in the elderly. Sarcopenia is a progressive decline in muscle mass and strength. One of the risk factors in this process is mitochondrial reactive oxygen species and oxidative stress. In a recent study, we tested the causal effect of targeting hydrogen peroxide within mitochondria to illuminate the role of mitochondrial oxidative stress in sarcopenia [1]. To specifically target hydrogen peroxide derived from the mitochondria, the expression of peroxiredoxin 3 (PRDX3) was 2–3 folds elevated in a transgenic mouse model specific to skeletal muscle (mPRDX3Tg). Peroxiredoxin 3 is a hydrogen peroxide scavenger localized within mitochondrial matrix [2]. The enzyme is also shown to scavenge greater than 90% of the hydrogen peroxide within the mitochondria due to its rich expression [3].

The findings in the study demonstrate that scavenging mitochondrial hydrogen peroxide improves mitochondrial functions that are critical for cellular respiration and calcium homeostasis. The decreased OXPHOS capacity in mice lacking superoxide dismutase 1 (Sod1KO) [4] was fully recovered in mPRDX3Tg mice. Our findings are consistent with a report using a similar model, mitochondria targeted overexpression of catalase in muscle (mMCAT) [5]. Calcium retention capacity of the mitochondria was also elevated in both transgenic models. Another important calcium regulator during contractile activities is sarco-endoplasmic reticulum ATPase (SERCA). Both mPRDX3Tg and mMCAT models attenuated downregulation of SERCA pump activity in Sod1KO mice. The protective mechanisms against mitochondrial defects in the Sod1KO model remains unclear, but mitochondrial biogenesis does not seem to be the contributing factor in mPRDX3Tg mice [1]. The mechanisms by which mitochondrial oxidative stress is linked to other functions is still unclear and is warranted for future investigations.

Disruption at neuromuscular junction is widely considered as one of the key drivers of sarcopenia. An important question in this process is the role of oxidative stress from mitochondria. Animals with nerve transection surgery that results in denervation exhibit 30–40 folds increase in reactive oxygen species from skeletal muscle mitochondria [6]. This data demonstrates the potential role of oxidative stress in the connection between muscle and nerve. We asked whether scavenging muscle derived hydrogen peroxide has a retrograde effect and protects against neuromuscular junction disruption. The PRDX3Tg mice demonstrated no evidence of such an effect [1]. On the contrary, the mMCAT mice were fully protected against acetylcholine fragmentation and denervation of the Sod1KO mice [5]. The reasons for the discrepancy are unclear, but one possibility is involvement of other antioxidants and cofactors in the peroxiredoxin system, including thioredoxin, thioredoxin reductase and NADPH. Recently, mitochondria-localized thioredoxin 2 has been shown to protect against sarcopenia associated with its impact on anti-apoptosis [7]. It would be important to overexpress multiple antioxidant enzymes involved in the peroxiredoxin and thioredoxin system and test their combined overexpression in sarcopenia.

Targeting mitochondrial hydrogen peroxide attenuates muscle atrophy and contractile dysfunction in a mouse model of early onset of sarcopenia [1, 5]. Atrogin1 and MuRF1 expressions are E3 ligases involved in protein degradation. These enzymes are elevated in diseases and pathological conditions that drive muscle wasting and atrophy. PRDX3 overexpression abrogated upregulation of MuRF1. Muscle fiber cross-sectional area was also increased in mPRDX3 mice. Part of the protective effect of PRDX3 on force generation is presumably the prevention and reduction of oxidative damage to contractile proteins or calcium regulatory proteins. Targeting mitochondrial hydrogen peroxide shortens the time-to-half-relaxation (1/2 RT) after twitch contraction, indicating improvement in calcium reuptake mechanisms. These findings are consistent with the improved SERCA pump activity [1]. In addition, mitochondrial hydrogen peroxide might be involved in other aspects of calcium handling (i.e., calcium release). Using a whole body MCAT mice, Umanskaya et al. demonstrated that improvement of calcium release by stabilizing calstabin in age-associated sarcopenia [8].

In conclusion, recent evidence using various transgenic mouse models clearly illustrates the potential of targeting mitochondrial oxidative stress in prevention and attenuation of sarcopenia. These animal models have increased antioxidant enzymes within skeletal muscle but targeting neuronal mitochondrial hydrogen peroxide might also protect against neuromuscular junction disruption and neurogenic atrophy as an independent mechanism. Future research should narrow our targets for interventions and pharmacological therapies that can help increase active and healthy lifespan in the elderly.
